# Formation mechanism and functional properties of walnut protein isolate and soy protein isolate nanoparticles using the pH-cycle technology

**DOI:** 10.3389/fnut.2023.1135048

**Published:** 2023-02-10

**Authors:** Yixin Dai, Ying Xu, Chunhe Shi, Ye Liu, Shuang Bi

**Affiliations:** Beijing Engineering and Technology Research Center of Food Additives, Beijing Advanced Innovation Center for Food Nutrition and Human Health, School of Food and Health, Beijing Technology and Business University, Beijing, China

**Keywords:** walnut protein isolate, soy protein isolate, pH-cycle, nanoparticles, interaction, functional properties

## Abstract

Walnut protein isolate (WPI) is a nutritious protein with poor solubility, which severely limits its application. In this study, composite nanoparticles were prepared from WPI and soy protein isolate (SPI) using the pH-cycle technology. The WPI solubility increased from 12.64 to 88.53% with a WPI: SPI ratio increased from 1: 0.01 to 1: 1. Morphological and structural analyses illustrated that interaction forces with hydrogen bonding as the main effect jointly drive the binding of WPI to SPI and that protein co-folding occurs during the neutralization process, resulting in a hydrophilic rigid structure. In addition, the interfacial characterization showed that the composite nanoparticle with a large surface charge enhanced the affinity with water molecules, prevented protein aggregation, and protected the new hydrophilic structure from damage. All these parameters helped to maintain the stability of the composite nanoparticles in a neutral environment. Amino acid analysis, emulsification capacity, foaming, and stability analysis showed that the prepared WPI-based nanoparticles exhibited good nutritional and functional properties. Overall, this study could provide a technical reference for the value-added use of WPI and an alternative strategy for delivering natural food ingredients.

## 1. Introduction

Recently, plant proteins have been applied as raw materials for nanoparticles due to several advantages, including easy surface modification, good biodegradability, biocompatibility, high targeting, and environmental protection (1). Some studies described that nanoparticles produced with proteins derived from plants are being widely used for the encapsulation and delivery of biomolecules, leading to an enhancement in the stability of nutrients and food shelf life (2–4).

It has been described that walnuts are a crucial source of proteins, and walnut protein isolate (WPI) has a high digestibility (85%) and a reasonable proportion of essential amino acids (5–7). In addition, some studies demonstrated that WPI exhibits anti-inflammatory, antioxidant, and blood pressure-lowering activities (5, 8). However, WPI contains a large amount of glutenin (more than 70%), which leads to its low water solubility. The poor solubility of walnut limits its application of walnut in high value-added fields (9). Furthermore, the low solubility of WPI has been highly associated with its high surface hydrophobicity (10). For instance, hydrophobic regions commonly provide enough binding sites for many biological molecules (11). Therefore, proteins presenting low solubility and high surface hydrophobicity (such as zein, glutenin, gliadin, and rice proteins) were crucial raw materials for nanoparticles (12). Previous studies have shown that for insoluble proteins, it was possible to form nanoparticles by complexing other macromolecules, thus increasing the solubility of insoluble proteins and expanding their applications (13). Over the years, some researchers successfully prepared stable protein-based composite nanoparticles using zein and sodium caseinate as the raw material (14). In addition, some reports revealed that protein-based composite nanoparticles were prepared from hydrophobic rice protein and hydrophilic pea protein, or whey protein (15, 16), increasing the solubility of rice protein. Therefore, WPI could be useful as a nanoparticle component.

Moreover, the presence of other proteins, such as soy protein isolate (SPI), is also essential for preparing composite nanoparticles from insoluble proteins, including WPI. Due to the good biocompatibility and bioavailability of SPI (12), a previous study successfully obtained protein based composite nanoparticles containing scallop muscle protein and SPI, and the solubility of scallop muscle protein was significantly improved (17). Additionally, the other study prepared protein-based composite nanoparticles using rice protein and SPI, and a significant increase in the functional properties of the protein-based composite nanoparticles was detected compared to original rice protein. The solubility of rice protein in nanoparticles was enhanced (18). A recent study also demonstrated that protein-based composite nanoparticles prepared from wheat gluten protein and SPI presented an increase in the solubility of wheat gluten protein and its nutritional properties (19). Therefore, SPI could be useful as a nanoparticle component.

Numerous studies have focused on structural modifications of walnut proteins to improve their solubility. Based the purpose, several methods have been applied, including physical (20), chemical (21), and biological (22). However, physical methods consume a large amount of energy during the process, which results in high costs. For chemical methods, they exhibit potential safety hazards, and for biological methods they lead to a decrease in the nutritional, functional, and sensory properties of proteins. The pH-cycle technology is a low-energy, organic solvent-free, and simple method to prepare nanoparticles. In this technology, a non-covalent interaction of two biomacromolecules occur (by adjusting the pH of the environment in which the two biomacromolecules are located) resulting in the burial of hydrophobic and the exposure of hydrophilic groups to obtain hydrophilic nanoparticles stable in neutral water systems (23). In previous studies, the pH-cycle technology overcomes the poor solubility of insoluble proteins and improves the biological functions after the co-assembly of proteins obtained from different sources into hydrophilic nanoparticles (14). The pH-cycle technology could improve the solubility of insoluble proteins and maintain the protein structure compared to the traditional methods described above, and this technology could help to retain nutritional properties and physiological activities with low damage to nutrients, meeting the needs of green, low-cost, easy operation, high-efficiency, safety, and has high commercialization potential (24). Since WPI contains many hydrophobic groups, in this study, the pH-cycle technology was performed to treat walnut proteins to obtain nanoparticles and eventually improve their functional properties. To date, the interaction mechanism between WPI and SPI-forming nanoparticles under the pH-cycle technology remains unclear, and the applicability of nanoparticles prepared using WPI and SPI in bioactive ingredients delivery has not been investigated.

Therefore, this study had three main aims. Firstly, this study aimed to perform a co-assembly between WPI and SPI into hydrophilic nanoparticles to enhance the WPI water solubility. Secondly, this study also tried to understand potential alterations in the protein structure and investigate the mechanism of interaction between WPI and SPI. Finally, several biological properties of the protein nanoparticles, including the foaming and emulsification, were measured, and the amino acid composition was determined to assess the performance of their functional and nutritional properties. This research has a broad prospect of contributing to the development of flavor-enhanced beverages and functional beverages with high nutritional value and helping the development of beverage processing industry. Overall, this study could help to develop soluble hydrophilic nanoparticles and expand their application in the commercial food industry. Furthermore, this research work could provide a new strategy for the solubilization of hydrophobic plant proteins, which can improve their applicability and acceptability in the industry and among consumers.

## 2. Materials and methods

### 2.1. Materials

In this study, the walnuts were purchased from the Winsuk County Woody Grain and Oil Forestry (Xinjiang, China). The soybean meal was acquired from Shandong Yuwang Group (Shandong, China). The 2 × Laemmli sample buffer, colored pre-stained proteins molecular weight standards, and pre-prepared gels were purchased from Shanghai Biyuntian Biotechnology Co., Ltd. (Shanhai, China). Methanol, ethanol and acetonitrile were purchased from MREDA Technology Co., Ltd. (HPLC grade, Beijing, China). Hydrochloric acid (HCl) and sodium hydroxide (NaOH) were purchased from Sinopharm Chemical Reagent Co., Ltd (analytical grade, Shanghai, China).

### 2.2. Sample preparation

#### 2.2.1. Extraction of WPI

In this study, WPI was prepared according to a previous methodology with slight modifications (25). Briefly, the walnut kernels were degreased using a customized oil press (Shandong Changjian Hydraulic Equipment Co., Ltd., Shandong, China), and then mixed with a hexane solution (1:10, w/v) overnight. After drying, the defatted walnut meal was crushed using a JYS-M01 grinder (Jiu Yang Co., Ltd., Shandong, China) and passed through a 100-mesh sieve. Then, the powder was mixed with ultrapure water (1:40, w/v), and the pH of the mixture was adjusted to pH 12 with 1 M NaOH and stirred for 2 h at room temperature. Obtained samples were centrifuged (HITACHI CR220, Tokyo, Japan) at 10000 × *g* and 4°C for 20 min. After centrifuging the supernatant was collected. After the pH of the supernatant was adjusted to pH 4.5 by 1 M HCl, and centrifuged at 10,000 × *g* and 4°C for 20 min to collect the precipitates. Finally, the precipitates were washed three times with ultrapure water (adjusted to pH 7 and dialyzed for 48 h). The samples were dried using an Alpha 2–4 Freeze Dryer (Christ, Osterode, Germany) to obtain powdered WPI. The proteins and moisture contents of WPI were 89.56 ± 0.48% (N × 5.3) and 4.24 ± 0.03%, respectively.

#### 2.2.2. Extraction of SPI

In this study, the SPI was prepared following a previous method with slight modifications (26). Briefly, soybean meal was mixed overnight with hexane (1:10, w/v) for degreasing. After the defatted soybean meal was crushed and sieved through 100 mesh. After, the powder was mixed with ultrapure water (1:20, w/v), the pH of the mixture was adjusted to pH 9 with 1M NaOH, stirred for 2 h at room temperature, and after centrifugation at 10,000 × *g* and 4°C for 20 min, the supernatant was collected. Then, the pH of the supernatant was adjusted pH 4.5 by 1 M HCl, and centrifuged at 10,000 × *g* and 4°C for 20 min to collect the precipitates. The precipitates were washed three times with ultrapure water (adjusted to pH 7 and dialyzed for 48 h), and freeze-dried to obtain a SPI powder. Finally, the proteins and moisture contents of SPI were 92.48 ± 0.26% (N × 6.25) and 4.6 ± 0.05%, respectively.

#### 2.2.3. Preparation of composite nanoparticles with WPI and SPI

To prepare the composite nanoparticles, the SPI was dissolved in WPI suspensions (1% w/v), and several protein mixtures were obtained from different WPI/SPI ratios (WPI: SPI ratios between 1:0.01 and 1:1, w/w). Then, the pH of the protein mixture was adjusted to 12 using 1 M NaOH to ensured full dissolution. After magnetic stirring for 1.5 h, the pH of the protein mixture was adjusted to 7 using 0.1 M HCl, and the solution was centrifuged at 5000 × *g* for 10 min to collect the supernatant and precipitate. Next, the supernatant was dialyzed for 24 h to obtain solution of composite nanoparticles. A part of solution was kept and used in each parameter analysis, and the remaining solution was lyophilized to perform a sodium dodecyl sulfate-polyacrylamide gel electrophoresis (SDS-PAGE) and amino acid analyses. On the other hand, the precipitate was lyophilized to do the SDS-PAGE and solubility evaluation. Unless otherwise specified, solutions of composite nanoparticles, WPI and SPI were used for the samples used in the subsequent determination of each parameter.

### 2.3. SDS-PAGE analysis

In this study, analysis of protein subunits by SDS-PAGE. The SDS-PAGE analysis was performed according to a previous report with slight modifications (27). In this study, the samples were prepared in a loading buffer containing 0.1 M Dithiothreitol (DTT) and incubated in a water bath at 95°C for 10 min. The electrophoretic gel was run at 120 V for 80 min after 10 uL of sample was loaded to electrophoretic gel. Then, the electrophoretic separation gel was stained with a Coomassie brilliant blue staining solution for 120 min, and the excess dye solutions were removed with the Coomassie brilliant blue destaining solution. Finally, the protein bands were photographed by the chemical gel imaging system Bio-Rad (Bio-Rad, Hercules, USA).

### 2.4. Nitrogen solubility index (NSI)

In this study, the solubility of proteins was determined using the NSI. After preparing the samples (as described in the section “2.2. Sample preparation”), the nitrogen content of the precipitated fraction was determined using the Kjeldahl method (28). The protein conversion factor was 5.3 and 6.25 for WPI and SPI, respectively. According to the SDS-PAGE results, no significant bands were detected in the SPI precipitated. Therefore, the NSI only referred to the WPI solubility. The NSI of WPI was calculated using the following Eq. 1:


(1) $$N\,S\,I=\frac{P_{W\,P\,I}-P_{r\,e\,s\,i\,d\,u\,e}}{P_{W\,P\,I}}\times 100$$

Where *P*_WPI_ was the mass of initial WPI (g), *P*_*residue*_ was the mass of residual protein (g).

### 2.5. Composite nanoparticles morphology

#### 2.5.1. Transmission electron microscopy (TEM)

In this study, the micromorphological observations of composite nanoparticles were performed by TEM. The samples were diluted in ultrapure water to reach a protein concentration of 0.02% (w/v). After, the sample was added dropwise to a copper grid, dried, and analyzed using the Jem 2100F TEM (Jeol, Tokyo, Japan).

#### 2.5.2. Atomic force microscopy (AFM)

In this study, the micromorphological observations of composite nanoparticles were performed by AFM. The samples were diluted with ultrapure water to obtain a final protein concentration of 0.0002% (w/v). After, 2.5 μL drops were placed on mica sheets and dried overnight at room temperature. Finally, the samples were used for the 5000II AFM (Hitachi, Tokyo, Japan) analysis.

### 2.6. Dynamic light scattering (DLS)

In this study, the particle size of composite nanoparticles were analyzed by DLS. DLS measurements were carried out by Nano-ZS90 (Malvern, Malvern, UK). The samples were diluted in ultrapure water to a final protein concentration of 0.1% (w/v) and were analyzed at 25°C. The refractive indices of the proteins and the dispersion medium were 1.450 and 1.330, respectively.

### 2.7. Fluorescence chromatography

In this study, the analysis of protein-protein interactions were performed by fluorescence chromatography. The samples were adjusted to a final protein concentration of 0.1% (w/v) with ultrapure water, and the intrinsic fluorescence spectra of complex nanoparticles were measured at room temperature using a FS5 fluorescence spectrometer (Edinburgh Instruments, Livingston, UK). An excitation wavelength of 280 nm was applied in this study, and the emission spectra was recorded between 300 and 400 nm. The theoretical fluorescence (Theor.) spectra were obtained by adding the fluorescence spectra of individual WPI and SPI with different concentrations in the samples, on the other hand, the experimental fluorescence (Exp.) spectra corresponded to the fluorescence spectra of complexed nanoparticles (29). In this study, the significance of hydrogen bonding, hydrophobic interactions, and electrostatic interactions in the interaction between WPI and SPI was also evaluated. For that, WPI and SPI were dissolved in 36 mL of distilled water and processed according to the methodology described in the section “2.2. Sample preparation.” Then, 4 mL of 0.1 M thiourea, SDS, and NaCl solutions (with a final concentration of 10 mM) were added to the protein samples. Next, the fluorescence spectra were collected using the same methodology used to prepare the previous samples.

Structural studies on composite nanoparticles were performed using the fluorescent probe ANS. This probe can specifically bind to the hydrophobic domain of the protein, being capable of detecting changes in the protein microenvironment. Briefly, the samples were diluted to a final protein concentration of 0.05% (w/v), then 10 μL of 8 M ANS was added to 4 mL of sample, and the reaction was completed kept in the dark for 15 min. Next, the samples were excited at 390 nm wavelength, and the fluorescence intensities were measured between 400 and 600 nm.

### 2.8. Circular dichroism (CD)

In this study, the analysis of protein secondary structure was performed by CD spectra. To determine the CD spectra of composite nanoparticles, a MOS-450 spectrometer (BioLogic Science Instruments, Ltd., Claix, France) was used. In this study, the samples were collected at different pH values during the pH-cycle process and diluted with ultrapure water. The wavelength range of far-UV CD was obtained between 190 and 250 nm, and the protein concentration was 0.05% (w/v). The near-UV CD was composed of wavelengths between 250 and 320 nm, and the protein concentration used was 0.01% (w/v). Distilled water was used as a blank solution for the determination.

### 2.9. Zeta-potential

In this study, the stability of composite nanoparticles were analyzed by zeta-potential. Zeta-potential measurements were carried out by Nano-ZS90 (Malvern, Malvern, UK). For this study, the samples were adjusted in ultrapure water to a final protein concentration of 0.1% (w/v) and analyzed at 25°C. The refractive indices of the proteins and the dispersion medium were 1.450 and 1.330, respectively.

### 2.10. Surface hydrophobic (H_0_)

In this study, the stability of composite nanoparticles were analyzed by the H_0_. The H_0_ parameter was determined using the ANS fluorescent probe method (30). The samples were diluted with ultrapure water to obtain different protein concentrations ranging between 0.0125 and 0.1% (w/v). After 4 mL of the samples were mixed with 10 μL of 8 mmol/L ANS solution and placed in the dark for 15 min. Then, the fluorescence intensity of composite nanoparticles was determined by FS5 fluorescence spectrometer at excitation wavelength of 390 nm and an emission wavelength of 484 nm. In this study, the H_0_ parameter was determined by the fluorescence intensity and protein concentration slope.

### 2.11. Amino acid analysis of composite nanoparticles

To determine the amino acid composition of the nanoparticles (g/100g protein), the proteins were hydrolyzed with hydrochloric acid. The co-assembled protein samples were placed into a sealed tube, and 10 ml of 6 mol/L hydrochloric acid solution was added. After neutralization with nitrogen, the tubes were sealed and hydrolyzed at 110 °C for 24 h. The supernatant was composed of a protein hydrolysate and determined by high-performance liquid chromatography. An Agilent 1200 liquid chromatograph equipped (Agilent Technologies Inc., Santa Clara, CA, USA) with a Zorbax Eclipse-AAA column (4.6 × 150 mm, 3.5 μm, Agilent Technologies Inc., Santa Clara, CA, USA) was employed to analyzing. The mobile phase was divided into phase A (pH 4.8, 0.04 mol/L NaH2PO4) and phase B (methanol: acetonitrile: ultrapure water (45:45:10 v/v/v)). The liquid phase conditions were as follows: flow rate, 1 mL/min; UV detection wavelength, 338 nm; injection volume, 1 mL; the column temperature was 40°C. The essential amino acid index (EAAI) was calculated according to formula 2 and corresponded to the geometric mean of the essential amino acid content and the corresponding amino acid content in the whole egg protein (31) (2):


(2) $$E\,A\,A\,I=100\times \sqrt[{n}]{\frac{L\,y\,s_{w}}{L\,y\,s_{s}}\times \frac{T\,h\,r_{w}}{T\,h\,r_{s}}\times \ldots \times \frac{V\,a\,l_{w}}{V\,a\,l_{s}}}$$

Where the subscript w indicates the protein sample, *s* indicates the whole egg protein, and *n* indicates the number of amino acids.

### 2.12. Functional properties of composite nanoparticles

#### 2.12.1. Emulsification capacity and stability of composite nanoparticles

In this study, the emulsification capacity and stability of composite nanoparticles were measured following a recent method with slight modifications (32). Briefly, the protein was mixed with deionized water to form a 1% (w/v) protein solution and stirred for 1 h to obtain complete hydration. Then, the protein solution was mixed with soybean oil to obtain an oil phase ratio of 0.25, followed by a 1 min homogenization at 20,000 r/min. After homogenization, 50 μL sample was mixed with 0.1% (w/v) SDS solution and diluted 100-fold. The UV absorbance of samples were measured using a UV-Vis spectrometer (Shimadzu UV-2600, Kyoto, Japan) at 500 nm (defined as A_0_). The emulsifying activity index (EAI) was calculated using the following formula (3):


(3) $$E\,A\,I\,\left(m^{2}/g\right)=\frac{2\times 2.303\times A_{0}\times D\,F}{C\times \left(1-\alpha \right)\times 1000}$$

Where A_0_ was the absorbance of emulsions at 0 min, c was the concentration of the protein used (g/mL), α was the oil volume ratio of the emulsion, and DF was the dilution multiple.

After 10 min, the sample was aspirated in the same method and added to the SDS solution to determine the UV absorbance value (designed as A_10_). The emulsion stability index (ESI) was calculated using the following formula (4):


(4) $$E\,S\,I\,\left(m\,i\,n\right)=\frac{A_{10}}{A_{0}-A_{10}}\times 10$$

Where A_0_ was the absorbance of emulsions at 0 min, and A_0_ was the absorbance of emulsions after 10 min.

#### 2.12.2. Foaming and foaming stability of composite nanoparticles

In this study, the foaming and foaming stability of composite nanoparticles were evaluated using a previous report with slight modifications (33). Briefly, the protein was mixed with deionized water to form a 1% (w/v) protein solution and stirred for 1 h to obtain complete hydration, and homogenized at 20,000 r/min for 1 min. Then, the foam volume was read and defined as V_0_. The homogenized sample was kept for 30 min, and after, the foam volume was measured again and defined as V_30_. Finally, the foaming capacity (FC) and foaming stability (FS) were calculated using the following formulas (5, 6):


(5) $$FC\left(\%\right)=\frac{V_{0}}{V}\times 100$$

(6) $$FS\left(\%\right)=\frac{V_{30}}{V_{0}}\times 100$$

Where V was the initial volume before foaming, V_0_ was the foam volume at 0 min after homogenization, and V_30_ was the foam volume at 30 min after homogenization.

### 2.13. Statistical analysis

At least three independent replicate experiments were performed for each determination of the sample to obtain the mean. Statistical results were expressed as mean ± standard deviation (SD) and analyzed using SPSS 20.0 software. Significant differences (*p* < 0.05) between groups were determined by one-way ANOVA variance.

## 3. Results and discussion

### 3.1. SDS-PAGE

**FIGURE 1:**
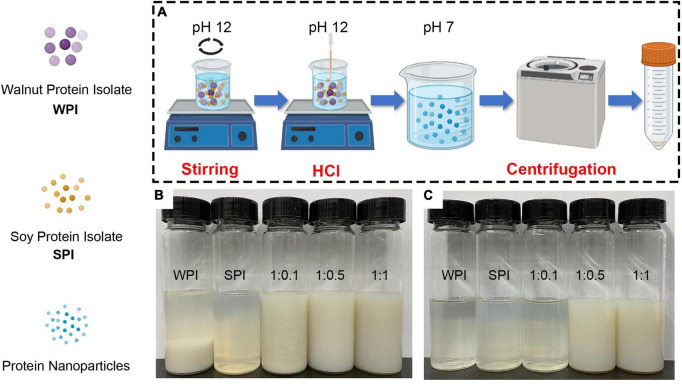
**(A)** Schematic illustration of the pH-cycle technique for fabricating protein-based composite nanoparticles with hydrophobic WPI and hydrophilic SPI. **(B)** Photographs of protein solutions and composite nanoparticles after the pH-cycle treatment. **(C)** Photographs of sample supernatants by centrifuging at 10,000 × *g* and 4°C for 20 min (1:0.1, 1:0.5, 1:1 represents walnut protein and soy protein isolate at the ratio of WPI:SPI = 1:0.1, 1:0.5, 1:1, respectively).

In this study, WPI and SPI were co-solubilized at pH 12.0 and then neutralized and collected to prepare the soluble composite nanoparticles (Figure 1A). The results demonstrated that WPI aggregated and precipitated at the bottom in an aqueous solution before the SPI addition (Figure 1B). However, after adding SPI and using the pH-cycle technology, the resulting composite nanoparticles could dissolve in water (Figure 1B). Furthermore, composite nanoparticles with WPI: SPI = 1:0.5 and 1:1 still maintained colloidal stability in neutral water systems (Figure 1C).

**FIGURE 2:**
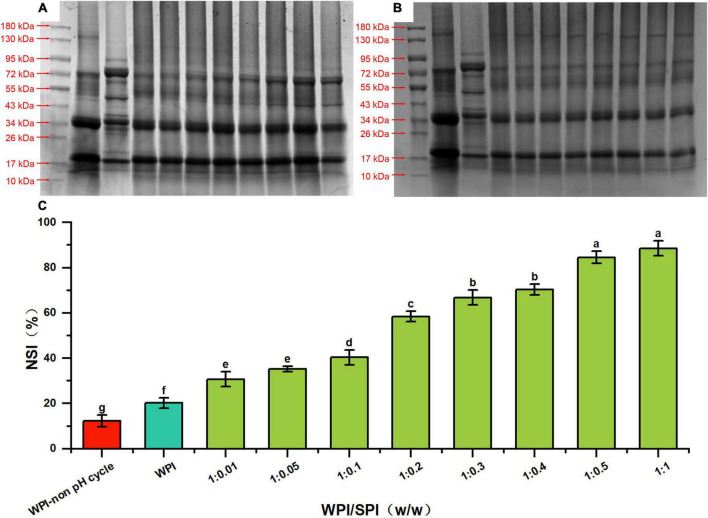
SDS-PAGE profiles of supernatant **(A)** and precipitates **(B)** of prepared composite nanoparticles, respectively (lane 1 is marker, lanes 2–10 are WPI, SPI, composite nanoparticles prepared by WPI: SPI = 1:0.01, 1:0.05, 1:0.1, 1:0.2, 1:0.3, 1:0.4, 1:0.5, and 1:1 (w/w), respectively). **(C)** Solubility of WPI in composite nanoparticles with WPI: SPI (w/w) from 1:0.01 to 1:1 by the pH-cycle technique (WPI represents walnut protein isolation solution without the pH-cycle treatment, control represents walnut protein isolation solution with the pH-cycle treatment).

As described above, the subunit information of the protein molecular weight was analyzed by the SDS-PAGE technology. The results obtained from the supernatants revealed that characteristic subunits of WPI and SPI were detected in composite nanoparticles. Furthermore, these subunit bands of SPI became more pronounced with the addition of SPI (Figure 2A, lanes 4–11). However, no characteristic subunit bands of SPI were observed in the SDS-PAGE performed with the precipitated fraction, indicating that only WPI was present after precipitation (Figure 2B, lanes 4–11). The composite nanoparticles in the supernatant retained the characteristic subunit bands of WPI and SPI after adding SPI and using the pH-cycle technology. These results suggest that most subunits of WPI and SPI were involved in the process of co-assembly to prepare composite nanoparticles and remained intact during the process. It has been described that conventional methods significantly degraded or removed most of the subunits of WPI, requiring a large quantity of energy and tedious labor and consequently leading to reduced applicability in the industry (34). This made the protocol significantly different from the conventional method. To investigate the changes in the solubility of walnut protein, the NSI was measured.

### 3.2. NSI

Studies described that protein solubility, a crucial functional property, could affect other several functional properties of proteins (35). The NSI of WPI in the composite nanoparticles was the percentage of WPI dissolved in solution relative to the initial WPI used in the reaction. Since the characteristic subunits of WPI were only present in the precipitate of composite nanoparticles (Figure 2B), the percentage of dissolved WPI can be obtained by calculating the difference between the total added amount and the content in precipitation. As shown in Figure 2C, the NSI of WPI with or without the pH-cycle treatment was approximately 20.09 and 12.64%, respectively. However, adding SPI induced an increase (30.71% at a WPI: SPI ratio of 1:0.01) in the NSI of WPI in the composite nanoparticles. Additionally, the NSI of WPI increased to 88.53% after adding SPI at a WPI: SPI ratio of 1:1, which was 7 times higher than in WPI nanoparticles without the pH-cycle treatment. Furthermore, the results showed that the WPI insolubility was significantly inhibited after the preparation of composite nanoparticles using the pH-cycle technology and adding SPI. On the other hand, most of the subunits in each protein were essentially unchanged in the composite nanoparticles (Figure 2A), demonstrating that the technology could improve the protein solubility and maintain molecular integrity. Since this study focused on WPI, SPI was added in an amount not exceeding WPI to avoid interferences with the structural analysis of composite nanoparticles. In this study, the morphological and structural properties of composite nanoparticles were evaluated to understand the formation mechanism of composite nanoparticles. The morphological of the composite nanoparticles were evaluated in the subsequent section.

### 3.3. Morphology of composite nanoparticles

**FIGURE 3:**
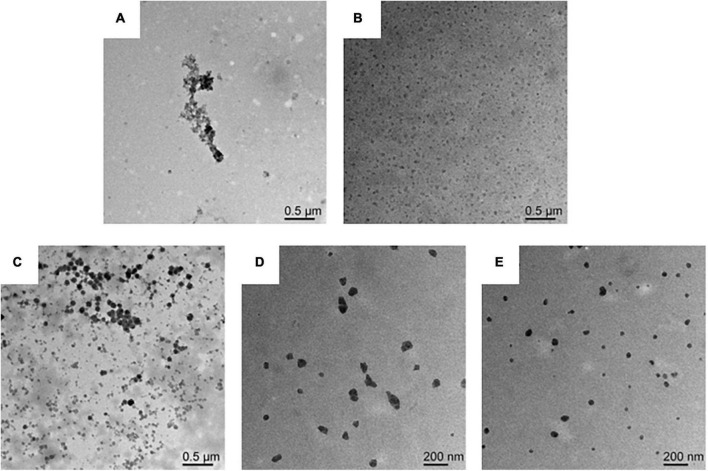
**(A)** Transmission electron microscopy (TEM) image of WPI solution. **(B)** TEM image of SPI solution. **(C)** TEM image of composite nanoparticles prepared by WPI: SPI (w/w) mass ratio of 1: 0.1. **(D)** TEM image of composite nanoparticles prepared by WPI: SPI (w/w) mass ratio of 1: 0.5. **(E)** TEM image of composite nanoparticles prepared by WPI: SPI (w/w) mass ratio of 1: 1.

**FIGURE 4:**
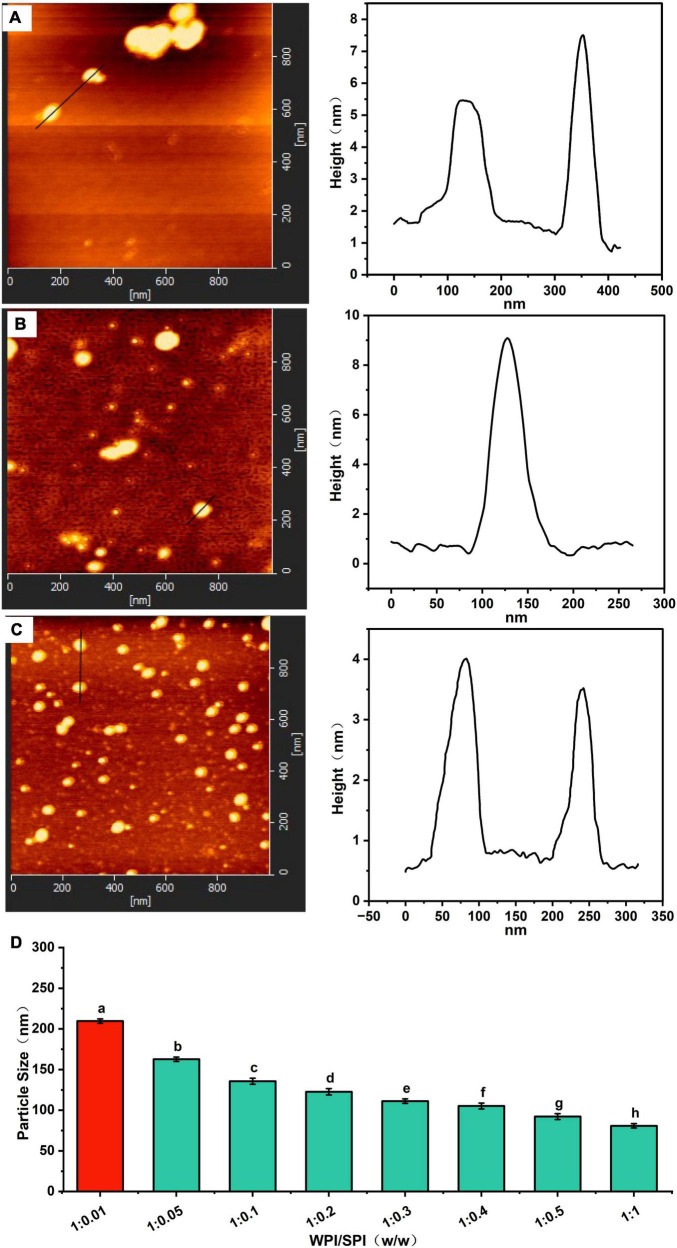
**(A)** Atomic force microscopy (AFM) image of composite nanoparticles prepared by WPI: SPI (w/w) mass ratio of 1: 0.1. **(B)** AFM image of composite nanoparticles prepared by WPI: SPI (w/w) mass ratio of 1: 0.5. **(C)** AFM image of composite nanoparticles prepared by WPI: SPI (w/w) mass ratio of 1: 1. **(D)** Particle size of composite nanoparticles with WPI: SPI (w/w) from 1:0.01 to 1:1.

The microscopic morphology of the protein and the composite nanoparticles can be visualized by TEM analysis. A significant aggregation was observed in TEM due to the high hydrophobicity of WPI (Figure 3A). Furthermore, an irregular aggregation was detected, leading to low protein solubility at pH 7.0, consistent with previous studies (36). On the other hand, SPI was well-dispersed in solution with uniform particle sizes at pH 7.0 (Figure 3B). Interestingly, the aggregation of proteins was reduced in the presence of the WPI/SPI = 1:0.1 ratio (Figure 3C). However, gradual, smaller, more dispersed, and homogeneous composite nanoparticles were obtained when the concentration of soy protein was enhanced (Figures 3D, E). To further investigate the structure of composite nanoparticles, AFM combined with the DLS technology was used. Therefore, the AFM results showed that an increase in SPI contributed to the dispersion of composite nanoparticles and the formation of uniform particles with a smaller size. These results agreed with the TEM results described above (Figure 4). The AFM results indicated that the composite nanoparticles collapse into nanoscale spherical structures on the mica sheet surface. The particle size of protein nanoparticles ranged between 80 and 250 nm, and a decrease was observed after adding SPI, which was in agreement with the TEM and AFM results (Figure 4D). Contrarily, the particle size was slightly larger than the size observed in TEM due to the crumpling of the sample that occurred during the drying pre-treatment in TEM. According to the microscopic and DLS analyses, it was possible to observe that when the two proteins interacted, a new protein structure was formed (30). To explore the mechanism behind the phenomenon of protein structure change, the structural properties of the composite nanoparticles were determined. The protein-protein interactions were elaborated.

### 3.4. Characterization of protein-protein interactions in composite nanoparticles

#### 3.4.1. Quenching of fluorescence of WPI by SPI in composite nanoparticles

**FIGURE 5:**
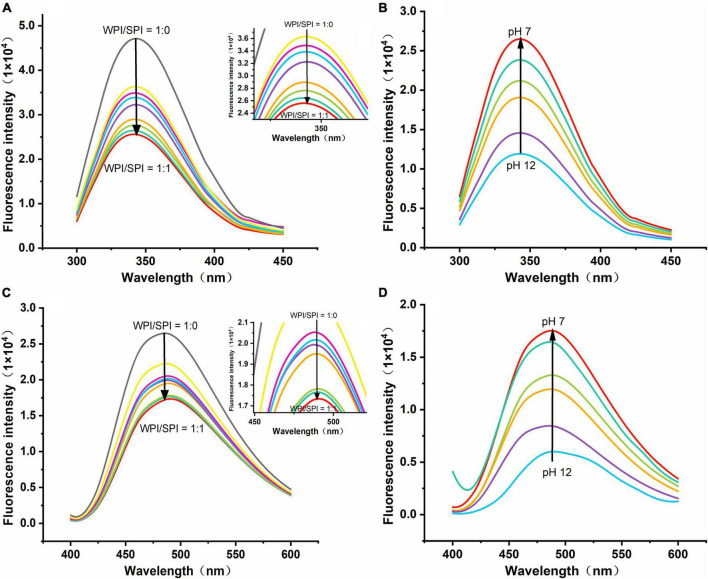
**(A)** Emission spectra of composite nanoparticles with WPI: SPI (w/w) from 1:0 to 1:1. **(B)** Emission spectra of composite nanoparticles (WPI: SPI = 1:1, w/w) at pH 12, 11, 10, 9, 8, and 7. **(C)** Emission spectra for ANS binding to composite nanoparticles with WPI: SPI (w/w) from 1:0 to 1:1. **(D)** Emission spectra for ANS binding to composite nanoparticles with WPI: SPI (WPI: SPI = 1:1, w/w) at pH 12, 11, 10, 9, 8, and 7.

In this study, to understand the interaction between WPI and SPI, fluorescence spectroscopy was performed. The WPI and SPI contained aromatic amino acids of chromogenic groups, such as tryptophan, phenylalanine, after excitation at 280 nm wavelength, these amino acids promoted the generation of intrinsic fluorescence (37). It has been described that protein interactions are accompanied by the quenching of endogenous fluorescence, while the quenched forms of different proteins reflect the changes in several regions and action sites (38, 39). Therefore, some reports suggest that these changes in endogenous fluorescence intensity can be used as indirect evidence about protein interactions. In the current study, the endogenous fluorescence intensity decreased, and fluorescence quenching occurred after adding SPI (Figure 5A). These results indicate that the interaction between WPI and SPI occurred and formed a new structure after adding SPI and using the pH-cycle technology. Moreover, the Exp. and Theor. spectra were compared, and the results are shown in Supplementary Figure 1. Some authors described that this phenomenon was related to the electron-rich aromatic amino acid groups toward the electron-deficient amino acid groups (40, 41). In this study, after adding SPI, a higher difference was observed between Exp. and Theor. For example, the degree of quenching increased, indicating that SPI was involved in the formation of the new structure (Supplementary Figure 1). Additionally, a gradual increase in the difference between Exp. and Theor. occurred when the pH decreased, mainly when the ratio of WPI and SPI was 1:1 (Supplementary Figure 2). This result suggests that the molecular affinity between WPI and SPI increased during the pH-cycle treatment, leading to an enhancement in protein interactions and, consequently, leading to the generation of a new protein structure. On the other hand, the maximum fluorescence intensity of the proteins increased when the pH decreased, indicating that the proteins underwent structural renaturation during the protonation process, leading to the formation of new structure (Figure 5B). Furthermore, increased fluorescence intensity was positively correlated with protein renaturation. Although the fluorescence intensity of the composite nanoparticles increased during the protonation process, this value remained lower compared to WPI, indicating that the structural renaturation of WPI could be inhibited by adding SPI (responsible for the increase in the WPI solubility).

In this study, the ANS probe was used to detect the folding and unfolding states of proteins since it can bind by hydrophobic interactions to the hydrophobic region of proteins, showing changes in microstructure and the surrounding environment polarity. For instance, after excitation at 390 nm wavelength, the fluorescence intensity of ANS can reflect the protein folding, and a higher intensity could indicate the higher degree of folding (42, 43). The present study demonstrated that the fluorescence intensity decreased after adding a higher concentration of SPI, indicating that the two proteins interacted and formed a new structure (Figure 5C). Therefore, this result suggests that an interaction between WPI and SPI occurred, and the resulting structure limits the acid-induced refolding of WPI due to increased rigidity, promoting the forming of a new hydrophilic rigid structure in a neutral environment. These conditions could significantly inhibit the formation of hydrophobic regions of the nanoparticles and promote a significant anti-folding behavior. Additionally, at pH 12, when the protein structure was maximally unfolded, the fluorescence intensity of the composite nanoparticles was minimal (Figure 5D). With the pH-cycle technology was performed, the pH was decreased, the increase in fluorescence intensity indicated an increase in the ANS binding sites, further demonstrating to protein refolding.

#### 3.4.2. Determination of type of interaction

**FIGURE 6:**
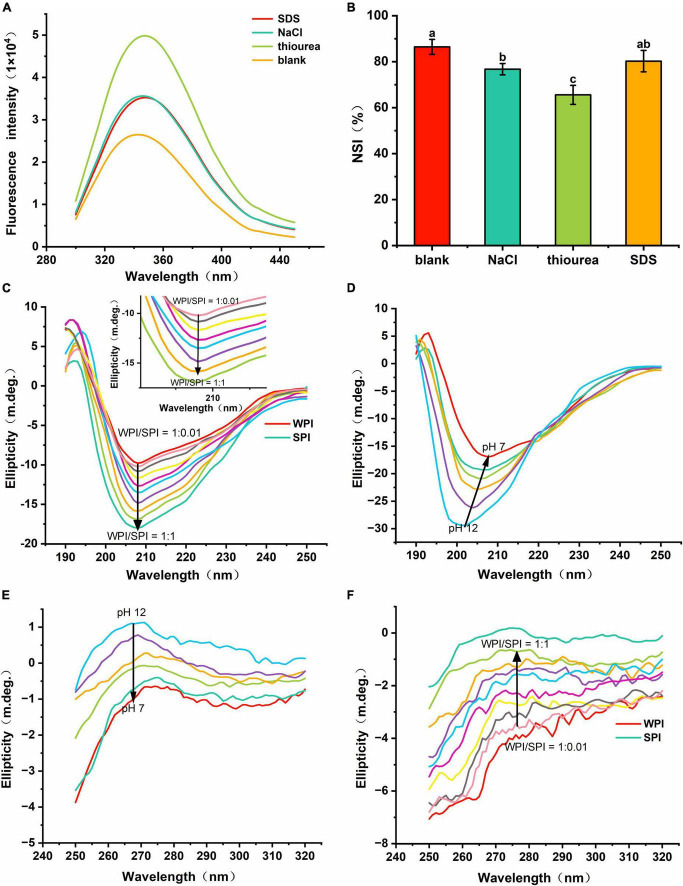
**(A)** Fluorescence spectra of composite nanoparticles WPI: SPI = 1:1 (w/w) with bond-disrupting agents added. **(B)** Nitrogen solubility index (NSI) of WPI with the addition of bond-disrupting agents (10 mM). **(C)** Far-UV CD spectra of composite nanoparticles with WPI/SPI (w/w) from 1:0.01 to 1:1. **(D)** Far-UV CD spectra of a representative composite nanoparticles (WPI: SPI = 1:1, w/w) prepared at pH 12, 11, 10, 9, 8, and 7. **(E)** Near-UV CD spectra of a representative composite nanoparticles (WPI: SPI = 1:1, w/w) prepared at pH 12, 11, 10, 9, 8, and 7. **(F)** Near-UV CD spectra of composite nanoparticles with WPI: SPI (w/w) from 1:0.01 to 1:1.

This study also tried to determine the non-covalent interactions that are responsible for the interactions between WPI and SPI. For that, the same molar concentrations of SDS, NaCl, and thiourea were individually added to the protein mixture at pH 12. The role of hydrophobic, electrostatic interactions, and hydrogen bonding to promote the process of building and maintenance of a hydrophilic rigid structure of composite nanoparticles was determined using fluorescence intensity (44). The effects of above the block reagents on the molecular interactions/bonds of proteins are listed in Supplementary Table 1. The effect induced by the block reagents was contrary to the fluorescence quenching caused by protein-protein interactions. Furthermore, the fluorescence intensity of the samples in the presence of block reagents was higher than composite nanoparticles, indicating that the three types of non-covalent interactions/bonds could contribute to the formation of a hydrophilic and rigid protein structure (Figure 6A). In addition, the thiourea exhibited the most pronounced result compared to the other bond-disrupting agents, suggesting that hydrogen bonding was crucial for stabilizing the composite nanoparticles. The NSI of WPI in soluble composite nanoparticles was also measured after adding above block reagents. The results revealed that the NSI was significantly lower after adding thiourea and NaCl (Figure 6B), i.e., the absence of hydrogen bonding and electrostatic interactions restricted the interaction between WPI and SPI, inhibiting the WPI solubilization. In addition, after adding SDS, a slight increase was observed in the NSI compared to the other two groups. This result suggests that SDS could act as a denaturant and inhibit the self-aggregation of WPI during the neutralization process.

#### 3.4.3. Circular dichroism spectroscopy

Some studies described that the presence of amides in the protein backbone could generate CD signals susceptible to structural changes in proteins (45). In addition, the far-UV CD spectra region (comprised between 190 and 250 nm) gives information related to the protein secondary structure. The results in Figures 6C, D showed that the two proteins formed a different structure, supporting the previous hypothesis regarding the formation of a new protein structure. After adding SPI, an increase in the intensity was detected at 208 nm. However, no significant red-shift or blue-shift was observed in these samples, which led to alterations in the structure of the composite nanoparticles and differed from that of the WPI and SPI. Thus, these results suggest that the interaction between WPI and SPI could alter the protein structure, leading to the formation of highly hydrophilic composite nanoparticles. Also, a shift in the red signal occurred during the protonation (between pH 12.0 and pH 7.0) (Figure 6D). The above results showed that the protein structure expanded and exposed more charged groups at pH 12.0, and the strong repulsion between the internal groups of the protein expanded and extended the protein, leading to the generation of a stable structure (46). In the process of the pH-cycle technology performed, the protein could re-fold and aggregate due to the reduction in the number of charged groups in the protein surface. However, an enhancement in the folding resistance in WPI occurred after adding SPI. Thus, the composite nanoparticles could form the stable new hydrophilic rigid structure, under neutral conditions.

Furthermore, the aromatic amino acid residues were altered by the microenvironment in the near-UV region of the CD profile, reflecting the changes in the tertiary structure of the protein (47). In the near-UV CD spectrum (ranging between 250 and 320 nm), the CD signal at 275–282 nm was derived from the circular dichroism of Tyr (48, 49). Therefore, the near-UV CD signal of Tyr gradually increased when the pH decreased, indicating that the proteins were refolded (Figure 6E) (30). In addition, the wavelength at the maximum CD model was blue-shifted, indicating that more Try was encapsulated into the hydrophobic environment, which again demonstrated the refolding of the proteins. Moreover, when SPI was added, the near-UV CD signal gradually improved, significantly inhibiting the refolding of WPI (Figure 6F). Therefore, the interaction between WPI and SPI enhanced the anti-folding ability of WPI, forming a high-intensity hydrophilic structure, conferring strong hydrophilicity. Then, the interfacial properties of the nanoparticles were evaluated to understand their dispersion.

### 3.5. Interfacial properties of the composite nanoparticles

**FIGURE 7:**
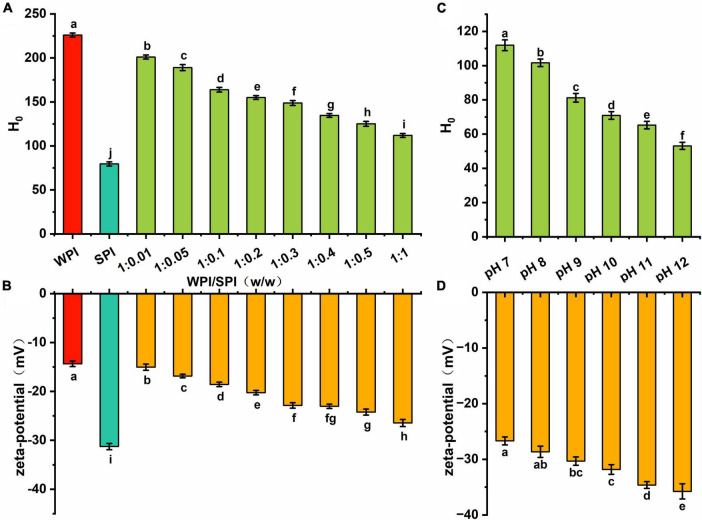
**(A)** The hydrophobicity of WPI, SPI and the composite nanoparticles at pH 7; **(B)** the zeta-potential of WPI, SPI and the composite nanoparticles at pH 7; **(C)** the hydrophobicity of the composite nanoparticles prepared at WPI: SPI ration of 1:1 at pH 12, 11, 10, 9, 8, and 7; **(D)** the zeta-potential of the composite nanoparticles prepared at WPI: SPI ration of 1:1 at pH 12, 11, 10, 9, 8, and 7.

Some studies revealed that substances with good water solubility must be self-repelling and inhibit aggregation. Therefore, the hydrophobicity can modulate their physical stability, solubility, aggregation tendency, and adsorption behavior (50). Due to the macromolecular nature of proteins, surface hydrophobicity can significantly alter the protein function compared to the total hydrophobicity (51). In the present study, surface hydrophobicity and zeta-potential were used to investigate the surface properties of the composite nanoparticles. The results in Figure 7A showed that the surface hydrophobicity of the composite nanoparticles decreased after adding SPI. However, an opposite trend was observed in the absolute zeta-potential values (Figure 7B). This result indicates that the interaction between the two proteins can lead to a gradual increase in the anti-folding of WPI, which continuously inhibited the continuously inhibits the formation of hydrophobic groups and the exposure of charged protein groups, creating repulsive force on the surface to improve the solubility and stability of the composite nanoparticles.

Additionally, this study investigated changes in the surface hydrophobicity and zeta-potential of the composite nanoparticles when the pH decreased. Figure 7C demonstrated that hydrophobic regions were continuously formed during the folding process. Furthermore, the surface hydrophobicity of composite nanoparticles was lower than in WPI, indicating that the interaction between WPI and SPI significantly inhibited the formation of hydrophobic regions and maintained the relative unfolding of the proteins conformation. The absolute values of zeta-potential continuously decreased with the decrease of pH, indicating that the charged groups exposed to pH 12 were encapsulated inside the complex structure (Figure 7D). The results showed that the decrease of surface hydrophobicity and the increase of net surface charge were crucial factors for good dispersion of the composite nanoparticles. Finally, the amino acid composition and functional properties were investigated.

### 3.6. Amino acid analysis of composite nanoparticles

WPI and SPI are considered excellent sources of amino acids for humans, being widely available in the diet (52), mainly WPI. The Food and Agriculture Organization of the United Nations and the World Health Organization (FAO/WHO) defined that nine essential amino acids are essential in dietary habits, including His, Ile, Leu, Lys Met + Cys, Phe + Tyr, Thr, Trp, and Val, to maintain normal body functions and health. For preschool children, the recommended levels of each amino acid are 19, 28, 66, 58, 25, 63, 34, 11, and 35 mg/g protein, respectively, and for adults, 16, 13, 19, 16, 17, 19, 9, 5, and 13 mg/g protein, respectively. The complex protein nanoparticles obtained in this study showed an impaired amino acid composition with EAAI values ranging from 71.73 to 74.61. On the other hand, SPI and WPI exhibited EAAI values of 70.72 and 78.25, respectively (Table 1). Therefore, it is possible to infer that the design of novel soluble protein complex structures composed of multiple proteins could be an effective strategy to obtain protein products with balanced nutritional composition and functionality.

**TABLE 1:** Amino acid composition of WPI-SPI nanoparticles.

Amino acid	WPI	SPI	WPI/SPI							
			1:0.01	1:0.05	1:0.1	1:0.2	1:0.3	1:0.4	1:0.5	1:1
GLU	22.28 ± 0.22^a^	20.25 ± 0.39^g^	22.06 ± 0.04^ab^	21.99 ± 0.06^ab^	21.79 ± 0.20^bc^	21.52 ± 0.28^cd^	21.54 ± 0.13^cd^	21.27 ± 0.11^de^	21.08 ± 0.18^ef^	20.82 ± 0.21^f^
ARG	14.80 ± 0.39^a^	8.72 ± 0.15^f^	14.20 ± 0.19^b^	13.89 ± 0.24bc	13.80 ± 0.18^bc^	13.65 ± 0.11^cd^	13.52 ± 0.27^cd^	13.31 ± 0.13^d^	12.88 ± 0.17^e^	12.59 ± 0.31^e^
ASP	9.57 ± 0.21^e^	13.36 ± 0.13^a^	9.67 ± 0.12^e^	9.87 ± 0.10^e^	9.78 ± 0.18^e^	10.70 ± 0.47^d^	11.02 ± 0.33^cd^	11.35 ± 0.15^bc^	11.65 ± 0.45^b^	11.52 ± 0.41^bc^
SER	5.37 ± 0.10^a^	4.16 ± 0.15^g^	5.33 ± 0.18^a^	5.21 ± 0.15^ab^	5.11 ± 0.01^bc^	5.05 ± 0.02^bc^	4.93 ± 0.06^cd^	4.82 ± 0.06^de^	4.72 ± 0.06^ef^	4.58 ± 0.06^f^
GLY	4.75 ± 0.11^a^	4.27 ± 0.11^bc^	4.69 ± 0.17^a^	4.71 ± 0.14^a^	4.63 ± 0.19^a^	4.59 ± 0.08^a^	4.55 ± 0.35^ab^	4.47 ± 0.11^ab^	4.28 ± 0.05^bc^	4.13 ± 0.09^c^
LEU	7.74 ± 0.19^d^	8.38 ± 0.32^a^	7.73 ± 0.05^d^	7.82 ± 0.03^cd^	7.84 ± 0.07^cd^	7.93 ± 0.11^cd^	8.04 ± 0.08^bc^	8.08 ± 0.07^bc^	8.21 ± 0.02^ab^	8.29 ± 0.22^ab^
PHE	4.89 ± 0.13^e^	6.32 ± 0.23^a^	4.95 ± 0.04^e^	5.35 ± 0.16^d^	5.72 ± 0.22^c^	5.75 ± 0.14^c^	6.05 ± 0.02^b^	6.13 ± 0.03^ab^	6.16 ± 0.08^ab^	6.24 ± 0.02^ab^
VAL	4.79 ± 0.11^a^	4.89 ± 0.11^a^	4.80 ± 0.14^a^	4.80 ± 0.15^a^	4.81 ± 0.01^a^	4.82 ± 0.18^a^	4.82 ± 0.06^a^	4.88 ± 0.11^a^	4.84 ± 0.03^a^	4.88 ± 0.09^a^
ALA	4.69 ± 0.11^a^	3.89 ± 0.05^c^	4.67 ± 0.13^a^	4.65 ± 0.06^a^	4.66 ± 0.19^a^	4.54 ± 0.15^ab^	4.49 ± 0.07^ab^	4.44 ± 0.06^b^	4.39 ± 0.03^b^	4.35 ± 0.08^b^
ILE	4.24 ± 0.23^a^	4.38 ± 0.22^a^	4.26 ± 0.12^a^	4.28 ± 0.08^a^	4.28 ± 0.19^a^	4.29 ± 0.11^a^	4.31 ± 0.15^a^	4.32 ± 0.07^a^	4.33 ± 0.22^a^	4.34 ± 0.02^a^
THR	3.79 ± 0.16^a^	3.65 ± 0.23^a^	3.75 ± 0.03^a^	3.75 ± 0.08^a^	3.73 ± 0.04^a^	3.71 ± 0.04^a^	3.66 ± 0.08^a^	3.65 ± 0.06^a^	3.64 ± 0.02^a^	3.63 ± 0.00^a^
TYR	3.41 ± 0.11^a^	3.46 ± 0.13^a^	3.41 ± 0.08^a^	3.42 ± 0.02^a^	3.42 ± 0.00^a^	3.42 ± 0.01^a^	3.43 ± 0.02^a^	3.43 ± 0.00^a^	3.43 ± 0.01^a^	3.42 ± 0.01^a^
LYS	2.53 ± 0.17^e^	6.22 ± 0.22^a^	3.67 ± 0.31^d^	3.71 ± 0.34^cd^	3.79 ± 0.07^cd^	3.83 ± 0.04^cd^	3.96 ± 0.47^bcd^	4.05 ± 0.12^bcd^	4.14 ± 0.05^bc^	4.29 ± 0.06^b^
HIS	2.48 ± 0.26^c^	2.85 ± 0.22^a^	2.44 ± 0.01^c^	2.48 ± 0.01^c^	2.53 ± 0.13^bc^	2.55 ± 0.32^bc^	2.55 ± 0.00^bc^	2.65 ± 0.01^abc^	2.73 ± 0.05^abc^	2.79 ± 0.10^ab^
MET	1.53 ± 0.18^a^	1.19 ± 0.12^c^	1.39 ± 0.14^ab^	1.34 ± 0.01^bv^	1.31 ± 0.02^bc^	1.34 ± 0.01^bc^	1.30 ± 0.01^bc^	1.23 ± 0.02^bc^	1.22 ± 0.08^c^	1.21 ± 0.05^c^
CYS	0.86 ± 0.03^a^	0.28 ± 0.02^e^	0.47 ± 0.01^b^	0.48 ± 0.00^b^	0.42 ± 0.01^c^	0.40 ± 0.06^c^	0.35 ± 0.01^d^	0.36 ± 0.01^d^	0.34 ± 0.01^d^	0.33 ± 0.00^d^
EAAI	70.72 ± 1.61^e^	78.25 ± 0.59^a^	71.73 ± 1.52^de^	72.34 ± 0.97^cde^	72.60 ± 0.97^cd^	72.93 ± 0.54^bcd^	73.15 ± 0.72^bcd^	73.57 ± 0.43^bcd^	73.89 ± 0.99^bc^	74.61 ± 0.57^b^

Values are expressed as the percentage weight of the amino acid against protein weight. Different superscript letters indicate significant differences (*p* < 0.05).

### 3.7. Functional properties of composite nanoparticles

Under external energy input, proteins can reduce the oil-water interfacial tension and induce the formation of emulsions in oil-water mixtures. Moreover, the emulsification performance was closely related to the hydrophilic-hydrophobic balance of the molecular structure (20). Therefore, solubility and hydrophobicity are crucial parameters to modulate the emulsification effect when proteins are used as interfacial stabilizers. The EAI and ESI of WPI were significantly lower than in SPI, demonstrating the limitation of the lack of solubility on its ability to adsorb at the oil-water interface (Supplementary Figures 3A, B). Additionally, the solubility and surface hydrophobicity of WPI was improved by the interaction with SPI, and the EAI and ESI of the composite nanoparticles were significantly higher than in WPI (Supplementary Figures 3A, B). Moreover, the co-assembled structure was relatively unfolded, and the structure was rearranged to expose the internal groups capable of adsorbing to the surface of oil droplets. Therefore, an improvement in the hydrophilic-lipophilic balance of the protein particles could occur, leading to an increase in the EAI in WPI (53). This result showed that solubility is highly relevant for protein emulsification. The increase in ESI occurred due to the interaction of WPI and SPI, resulting in the formation of a new three-dimensional network spatial structure. Therefore, the protein contains all the proprieties to adhere to the oil droplet’s surface. And the composite nanoparticles with a certain amount of surface charge are adsorbed to the oil-water interface to maintain the emulsion stability by the electrostatic repulsion between the interface-interface and then prevent the occurrence of oil droplet agglomeration (54).

Protein foaming properties can significantly alter the value for application in foamy foods, such as ice cream. Furthermore, some biological parameters, including protein solubility, molecular flexibility, hydrophobicity, and charge density, also significantly modulate the foaming ability (FA) (55). Additionally, changes in protein structure, size, and interactions could lead to alterations in the surface tension, viscoelasticity, and surface rheological properties and consequently affect the foaming stability (FS) (56). Taking this into account, the FA and FS parameters were evaluated in this study. The FA of the composite nanoparticles was significantly improved compared to WPI. However, the FS was boosted less (Supplementary Figures 2C, D). The results indicate that the interaction between WPI and SPI formed a new three-dimensional hydrophilic structure. The structure showed a relatively unfolded state, enhanced protein flexibility and solubility, and enhanced interaction between protein and water molecules, promoting protein adsorption at the liquid-air interface. All these changes could induce a significant improvement in the FA (57). Compared to WPI or SPI alone, the FS of the composite nanoparticles was enhanced because WPI and SPI interacted to form a new three-dimensional structure, exposing some of the hydrophobic groups. On the other hand, inter-protein forces were enhanced, resulting in rapid adsorption and formation of a stronger protein membrane at the air-water interface, enhancing the FS (55, 58). Overall, the composite nanoparticles showed improved functional properties compared to WPI or SPI alone.

## 4. Conclusion

In this study, soluble protein composite nanoparticles with a new hydrophilic rigid structure were successfully prepared by using pH cycling technology. The nanoparticles have an integrity of the protein primary structure. Furthermore, the structural changes between WPI and SPI were achieved by an interaction force with hydrogen bonding as the main effect, which significantly improved the anti-refolding property of protein, inhibited the acid-induce refolding, and formed a new high-strength hydrophilic structure. Under neutral conditions, the surface charged nature of the protein was maintained, resulting in an excellent water dispersion of the composite nanoparticles. Additionally, nutritional value and functional properties, such as EAI and FA of the proteins, were significantly enhanced during the pH-cycle treatment. Overall, this study can contribute to developing WPI-based nanoparticles with high nutritional value and functional properties. However, further studies are needed to evaluate the ability of the nanoparticles to improve bioavailability, control the essential oil release, and their applicability in the industry field.

## Data availability statement

The raw data supporting the conclusions of this article will be made available by the authors, without undue reservation.

## Author contributions

YD: conceptualization, methodology, investigation, and writing – original draft. YX: investigation and software. CS: investigation. SB: supervision. YL: project administration and funding acquisition. All authors contributed to the article and approved the submitted version.
