# Supplementary material for: Formation mechanism and functional properties of walnut protein isolate and soy protein isolate nanoparticles using the pH-cycle technology
Source: Front Nutr. 2023 Feb 10;10:1135048. doi: 10.3389/fnut.2023.1135048 (PMC9950265; doi:10.3389/fnut.2023.1135048)
Supplement: Supplementary file 1 [file Data_Sheet_1.PDF]

Supplementary Material

Formation mechanism and functional properties of walnut protein isolate and soy protein isolate nanoparticles using the pH-cycle technology

Yixin Dai, Ying Xu, Chunhe Shi, Ye Liu*, Shuang Bi*

*** Correspondence:** Ye Liu: liuyecau@126.com (Ye Liu); Tel: +86-13466390662; Full postal address: No. 11, Fucheng Road, Haidian District, Beijing, 100048, China. Phone/fax: +86-010-68985956. Shuang Bi: bishuang@btbu.edu.cn (Shuang Bi); Tel: +86-18810832420; Full postal address: No. 11, Fucheng Road, Haidian District, Beijing, 100048, China.

1 Supplementary Figures and Tables

1.1 Supplementary Figures

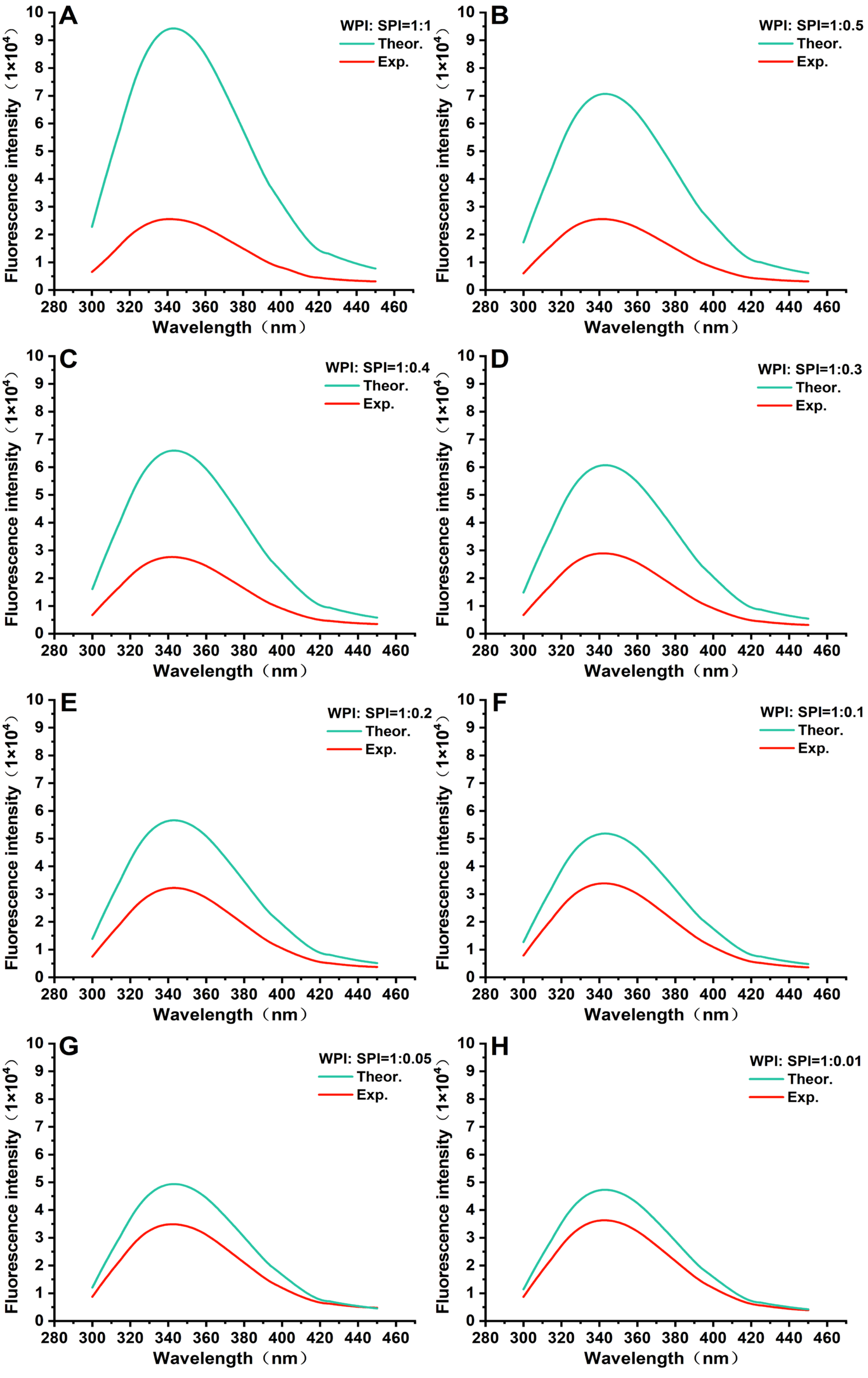
A
WPI: SPI=1:1
Theor.
Exp.
B
WPI: SPI=1:0.5
Theor.
Exp.
C
WPI: SPI=1:0.4
Theor.
Exp.
D
WPI: SPI=1:0.3
Theor.
Exp.
E
WPI: SPI=1:0.2
Theor.
Exp.
F
WPI: SPI=1:0.1
Theor.
Exp.
G
WPI: SPI=1:0.05
Theor.
Exp.
H
WPI: SPI=1:0.01
Theor.
Exp.
Fluorescence intensity（1×10⁴）
Wavelength（nm）
0 1 2 3 4 5 6 7 8 9 10
280 300 320 340 360 380 400 420 440 460

Supplementary Figure S1. Theoretical and experimental emission spectra of composite nanoparticles. (A) to (H) are composite nanoparticles prepared at WPI: SPI of 1:1, 1:0.5, 1:0.4, 1:0.3, 1:0.2, 1:0.1, 1:0.05 and 1:0.01, respectively.

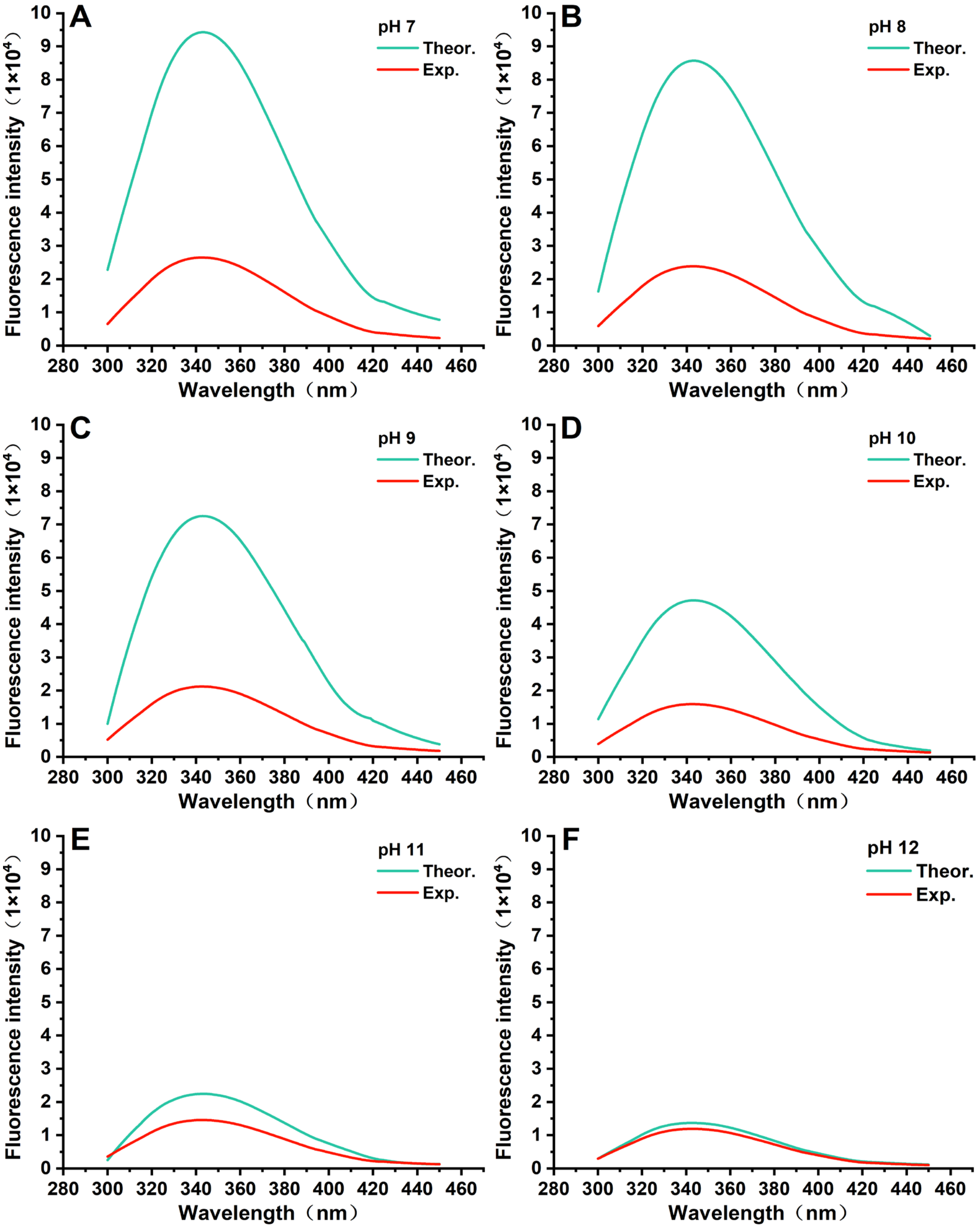

Supplementary Figure S2. Theoretical and experimental emission spectra of composite nanoparticles. (A) to (F) are composite nanoparticles (WPI: SPI=1:1, w/w) at pH 7, 8, 9, 10, 11 and 12, respectively.

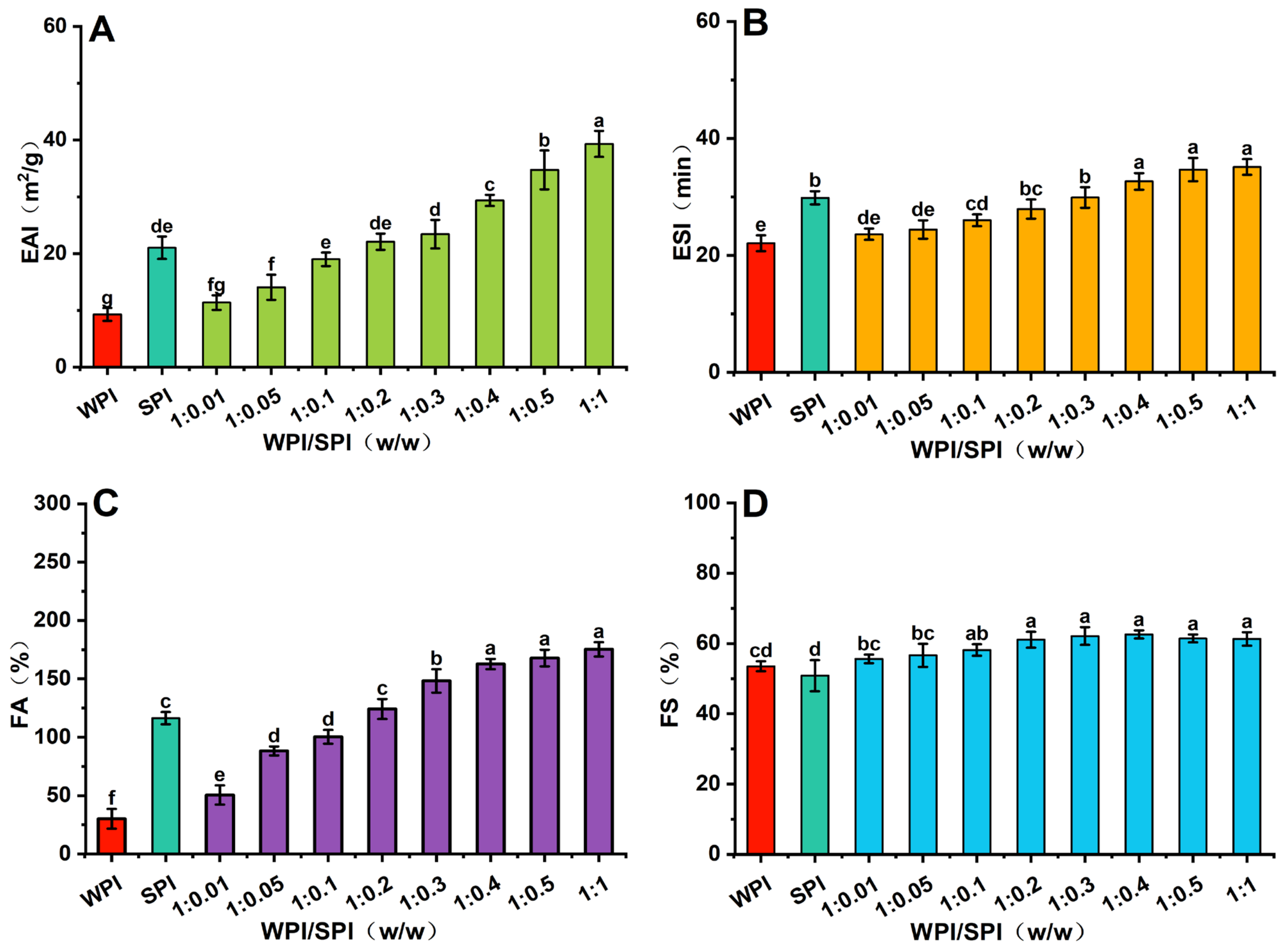

Supplementary Figure S3. (A) The emulsifying activity index and (B) emulsifying stability index of WPI, SPI and composite nanoparticles. (C) The foaming capacity and (D) stability of WPI, SPI and composite nanoparticles.

1.2 Supplementary tables

Supplementary table 1. Effects of various reagents on molecular interactions/bonds between WPI and SPI.

Type of reagents	Non-covalent interactions/bond	References
SDS	Hydrophobic interactions	Nilsson and Halle, (2005)
NaCl	Ionic effects/ electrostatic interactions	Nilsson and Halle, (2005)

Thiourea	Hydrogen bonding	Nilsson and Halle, (2005)
